# Long-standing diabetes mellitus increases concomitant pancreatic cancer risk in patients with intraductal papillary mucinous neoplasms

**DOI:** 10.1186/s12876-022-02564-8

**Published:** 2022-12-20

**Authors:** Atsushi Yamaguchi, Susumu Tazuma, Yuzuru Tamaru, Ryusaku Kusunoki, Toshio Kuwai, Hirotaka Kouno, Naoyuki Toyota, Takeshi Sudo, Kazuya Kuraoka, Hiroshi Kohno

**Affiliations:** 1 Department of Gastroenterology, Kure Medical Center and Chugoku Cancer Center, 737-0023, Aoyamacho 3-1, Kure, Hiroshima, Japan; 2 Department of Gastroenterology, Onomichi General Hospital, Onomichi, Hiroshima, Japan; 3 Department of Radiology, Kure Medical Center and Chugoku Cancer Center, Kure, Hiroshima, Japan; 4 Department of Surgery, Kure Medical Center and Chugoku Cancer Center, Kure, Hiroshima, Japan; 5 Department of Pathology, Kure Medical Center and Chugoku Cancer Center, Kure, Hiroshima, Japan

**Keywords:** Intraductal papillary mucinous neoplasm (IPMN), Pancreatic cyst, Pancreatic ductal adenocarcinoma (PDAC), Diabetes mellitus, Surveillance

## Abstract

**Background:**

When monitoring patients with an intraductal papillary mucinous neoplasm (IPMN), it is important to consider both IPMN-derived carcinoma and concomitant ductal adenocarcinoma (PDAC). The latter is thought to have a poorer prognosis. We retrospectively analyzed the risk factors for concomitant PDAC in IPMN.

**Methods:**

In total, 547 patients with pancreatic cysts, including IPMNs inappropriate for surgery on initial diagnosis, encountered from April 2005 to June 2019, were reviewed. We performed surveillance by imaging examination once or twice a year.

**Results:**

Five IPMNs with high-grade dysplasia and one IPMN associated with invasive carcinoma were encountered. In comparison, 14 concomitant PDACs were encountered. The prognosis was very poor for concomitant PDACs. All 14 PDAC patients had IPMNs. In patients with IPMNs, long-standing diabetes mellitus was the only significant risk factor for concomitant PDAC in both univariate and multivariate analyses (*P* < 0.001 and *P* < 0.01, respectively). Furthermore, patients with IPMNs and diabetes mellitus had a high frequency of concomitant PDACs (9.5%, 9/95) in a median 48-month surveillance period.

**Conclusions:**

When monitoring IPMNs, the development of not only IPMN-derived carcinomas but also concomitant PDACs should be considered. During this period, it may be prudent to concentrate on patients with other risk factors for PDAC, such as long-standing diabetes mellitus.

**Supplementary Information:**

The online version contains supplementary material available at 10.1186/s12876-022-02564-8.

## Background

Pancreatic ductal adenocarcinoma (PDAC) has the worst prognosis among cancers, and its 5-year survival rate is approximately 10% and 7.1% in the United States and Japan, respectively [1, 2]. Therefore, great effort has been made to detect pancreatic cancers at earlier stages, focusing on patients with risk factors for pancreatic cancer. Risk factors include hereditary pancreatic cancer syndrome, familial pancreatic cancer [3], chronic pancreatitis, and intraductal papillary mucinous neoplasm (IPMN) [3–7]. There are two pathways that promote pancreatic cancer in patients with IPMN: pancreatic cancer derived from IPMN and concomitant pancreatic cancer (PDAC) with IPMN. Concomitant PDAC develops at different site than that for IPMN, and its pathogenesis is still not well-understood. However, some clinical studies have shown that patients with IPMN had more PDACs (concomitant PDACs in this paper) than the general population [8–11]. Concomitant PDAC has been reported to be induced even after 5 years of surveillance, stressing the importance of vigilance in monitoring for concomitant PDAC with IPMN over a lengthy time course [8, 9]. The surveillance of patients diagnosed with IPMNs over many years might be difficult for patients and doctors and in terms of medical economics. The frequency of concomitant PDACs found during surveillance is very low compared with PDACs found for other reasons in our institute. For this reason, we need to focus on patients with a higher risk for pancreatic cancers, even in IPMN cohorts. Herein, we studied the risk factors for concomitant PDAC in a patient cohort with pancreatic cysts.

## Methods

This retrospective study included 547 patients diagnosed with a pancreatic cyst between April 2005 and June 2019 at the National Hospital Organization Kure Medical Center and Chugoku Cancer Center. We included patients who had imaging examinations at least once in a year and with a minimum of one year of routine imaging. We excluded the following: (1) cysts appropriate for therapy at initial diagnosis (symptoms from cyst, existence of mural nodule, main pancreatic duct [MPD] ≥10 mm, and jaundice); (2) cystic degeneration of known tumors (e.g., neuroendocrine neoplasm, solid pseudopapillary neoplasm, acinar cell carcinoma); (3) retention cysts from an obviously recognized tumor; and (4) pseudocysts accompanied with pancreatitis. This study was performed in accordance with the Declaration of Helsinki and was approved by our ethics committee (No. 2019-07). Patients were not required to give informed consent to the study because the analysis used anonymous clinical data that were obtained after each patient agreed to receive surveillance for pancreatic cysts. For disclosure, the details of the study are posted on some walls in the National Hospital Organization Kure Medical Center and Chugoku Cancer Center.

### Examination at initial diagnosis and follow-up

The height and body weight of patients were determined, and patients were interviewed regarding comorbidities (especially diabetes mellitus), a past history of malignancies, alcohol intake, smoking, and a family history (FH) of pancreatic cancer. Patients routinely underwent blood test for several items including blood glucose and hemoglobin A1c, abdominal contrast-enhanced computed tomography scans (CE-CT), magnetic resonance cholangiopancreatography (MRCP), and endoscopic ultrasonography (EUS) during their first visit to our hospital. This was usually followed by CE-CT, MRCP, or EUS twice a year. Using referral letters and self-reports, patients who were already taking medication for diabetes mellitus at initial diagnosis of a cyst were defined as patients with diabetes mellitus. In addition, patients who were diagnosed with diabetes mellitus in the wake of a blood test at the initial cyst diagnosis were also defined as patients with diabetes mellitus. Long-standing diabetes mellitus was defined as diabetes lasting for at least two years before the diagnosis of pancreatic cancer and new-onset was defined as lasting less than 2 years. We defined usual alcohol consumption as drinking over 20 g ethanol almost every day. A retrospective review of the collected data was performed for this study.

### Diagnosis of cyst type

First, we divided cysts into IPMNs, serous cystic neoplasms (SCNs), and non-IPMN/SCN cysts (Others). The diagnosis of IPMN was performed according to the 2017 international guidelines [7]. IPMN was defined as any cyst sized over five mm communicating with the MPD. We used mainly MRCP to ascertain the communication between MPD and cysts. SCN was diagnosed using CE-CT, MRCP, and EUS.

### Measurement of cyst diameter and main pancreatic duct diameter

We defined the max cyst diameter as the longest part on MRCP imaging. The MPD caliber was measured at the most dilated part that was not near the cyst in the MRCP.

### Further therapy

Further therapy, including surgical intervention, was offered to those suspected of having invasive pancreatic cancer or IPMN with high-grade dysplasia (HGD), based on imaging or histology and cytology. A cyst with a mural nodule or MPD ≥10 mm or positive cytology and concomitant PDAC was determined to be appropriate for further therapy.

### Differentiation of IPMN-derived carcinoma from concomitant PDAC

**Fig. 1:**
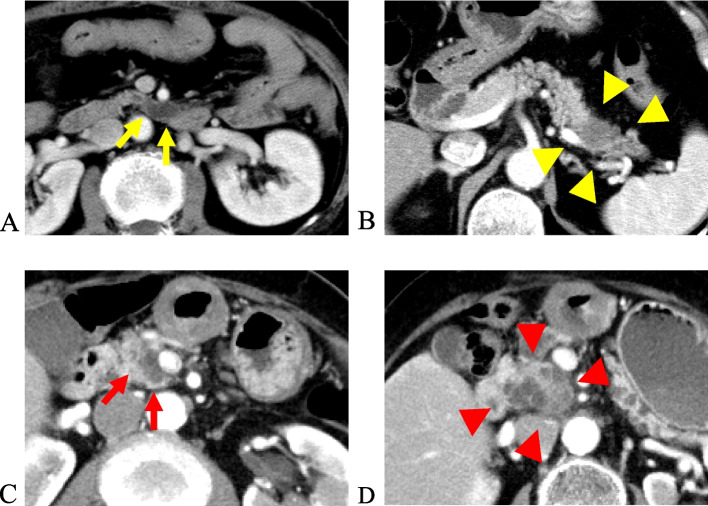
A patient was diagnosed with branch duct intraductal papillary mucinous neoplasm (BD-IPMN) involving the uncinate process of the pancreas (yellow arrows) (**a**), and concomitant pancreatic ductal adenocarcinoma (PDAC) developed in the pancreatic tail 51 months after initial cyst diagnosis (yellow arrowheads) (**b**). A patient was diagnosed with BD-IPMN involving the pancreas head (red arrows) (**c**) and later developed IPMN-derived PDAC (arrowheads) 66 months after initial diagnosis (red arrowheads) (**d**)

Concomitant PDAC is defined as occurring when the lesion is separated from the IPMN by an uninvolved segment of pancreatic duct, and there is no transition area from IPMN to carcinoma in the distinct PDAC [8, 12]. We first used surgical specimens, and imaging studies were used if surgery was not performed. Fig. 1 shows a patient with concomitant PDAC with IPMN (Figs. 1a, b) and a patient with an IPMN-derived carcinoma (Figs. 1c, d).

### Cumulative carcinogenic rate

The cumulative carcinogenic rates at 5 years and 10 years in patients with cysts were calculated via the Kaplan–Meier method.

### Standardized incidence ratio of pancreatic carcinoma

The standardized incidence ratio (SIR) of PDAC was calculated as the ratio of the observed versus expected number of patients who were diagnosed with PDAC. The expected number of PDAC cases was calculated using age-stratified and sex-specific data on the incidence of major cancer types in the general Japanese population, which was reported in 2017 by the Center for Cancer Control and Information Services, National Cancer Center [13].

### Statistical analyses

Fisher’s exact test was used to compare categorical variables, and the Welch’s *t-*test and Median test were used to compare quantitative data where appropriate. Logistic regression analysis was performed to identify independent predictors of the development of concomitant pancreatic cancer. The log-rank test with the Kaplan–Meier method was used to evaluate the risk of cancer in the univariate analysis, and a Cox regression hazard model was used for the multivariate analysis of the risk factors for pancreatic cancer. All statistical analyses of recorded data were performed using the Excel statistical software package (Ekuseru-Toukei 2015 version; Social Survey Research Information Co., Ltd., Tokyo, Japan). As for risk factors for development in concomitant PDAC, variables found to be possibly significant (*P*<0.15) by univariate analysis were chosen for entry into a multiple logistic regression. P-value for ‘IPMN or non-IPMN’ was 0.08 in univariate analysis, but we did not include this item for multivariate analysis because analysis was impossible due to multiplicity problem. In survival analysis, variables found to be possibly significant (*P*<0.15) by Kaplan–Meier method were chosen for entry into a Cox regression hazard model. P-value for ‘IPMN or non-IPMN’ was 0.06 in univariate analysis, but we did not include this item for multivariate analysis because analysis was impossible due to multiplicity problem. *P*<0.05 was considered as statistically significant.

## Results

### Patient characteristics

Table 1 summarizes the clinical characteristics of 547 patients with a pancreatic cyst. On initial diagnosis, there were 204 men and 343 women (37.3% and 62.7%, respectively) with a median age of 71 years (range, 31–93 years) and a median cyst size of 14.6 mm (range, 2–68). Cyst types were divided into IPMN (*n*=318), Others (*n*=99), IPMN+Others (*n*=120), and IPMN+SCN (*n*=1). The number of patients with IPMN was 439.

**Table 1:** Characteristics of patients

Number of patients	547
Year of diagnosis, n (%)	
2005 - 2009	85 (15.5)
2010 - 2014	250 (45.7)
2015 - 2019	212 (38.8)
Sex, M : F, n (%)	204 (37.3) : 343 (62.7)
Age, median (range), years	71 (31-93)
Maximum cyst diameter, median (range), mm	14.6 (2-68)
Cyst number, 1 : 2: ≥3	224 : 122 : 201
Diameter of main pancreatic duct, median (range), mm	2.5 (1-9.8)
IPMN	318 (58.1)
SCN	7 (1.3)
Others	99 (18.1)
IPMN + Others	120 (21.9)
IPMN + SCN	1 (0.2)
SCN + Others	2 (0.4)
Patients with IPMN, n (%)	439 (80.3)
Diabetes mellitus at cyst diagnosis, yes : no	112 : 435
History of malignancy, yes : no	152 : 395
Usual alcohol consumption (ethanol >20g/day), yes : no	161 : 386
Smoke (brinkman index ≥400) , yes : no	138 : 409
Family history of pancreatic cancer ≤1^nd^ degree, yes : no : N.A	41 : 502 : 4
Family history of pancreatic cancer ≤2^nd^ degree, yes : no : N.A	49 : 494 : 4
BMI (kg/m^2^) ≥30, yes : no	15 : 532
BMI (kg/m^2^) ≥25, yes : no	115 : 432

*M* male, *F* female, *IPMN* intraductal papillary mucinous neoplasm, *SCN* serous cystic neoplasm, Others is defined as non-IPMN and non-SCN cyst. BMI=body mass index

### Outcomes of follow-up

**Fig. 2:**
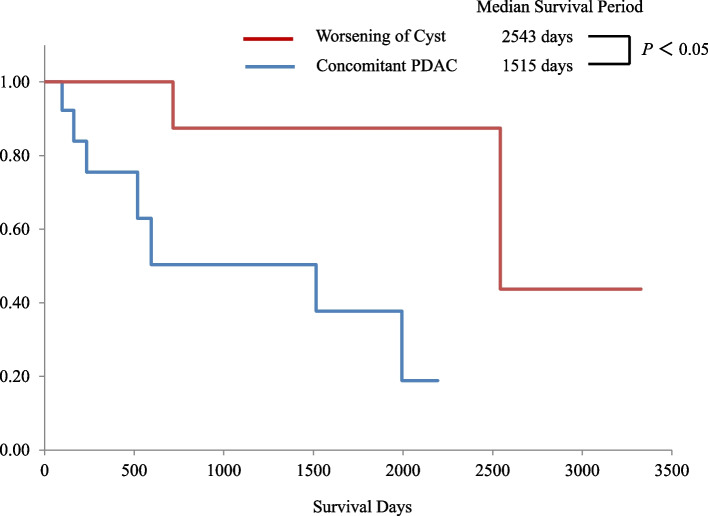
Survival curves. One of these curves (red) is from patients with further therapy from worsening of the cyst, and another one (blue) is from patients with concomitant pancreatic ductal adenocarcinoma (PDAC)

The results of the follow-up examinations are summarized in Table 2, and the details are shown in Supplementary Tables 1 and 2. At a median 59-month follow-up, 12 patients had further therapies due to worsening of a cyst (Supplementary Table 1). Nine patients were diagnosed using surgical specimens, and the other 3 patients were diagnosed with pancreatic juice cytology using endoscopic retrograde pancreatography and imaging examinations. There were 5 IPMNs with low-grade dysplasia (LGD), three IPMNs with HGD, two equivalent to IPMN with HGD (not resected case), one IPMN with associated invasive carcinoma (not resected case), and one SCN. There were 14 patients with concomitant PDAC, and the median duration to onset of concomitant PDAC was 45 months (14-119). Out of 14 patients, twelve patients were diagnosed using surgical specimens, and the other 2 patients were diagnosed with fine needle aspiration using EUS and imaging examinations. Fig. 2 shows the survival curves of patients with therapies for worsening cysts and concomitant PDACs. The median survival period was significantly worse in the latter than in the former (51 months vs. 85 months, *P*< 0.05).

**Table 2:** Outcome of follow-up for all patients with cyst

Number of patients	547
Follow-up period, median (range), months	59 (13 - 177)
Age at final examination, median (range), years	76 (32 - 99)
Further therapy due to worsening of cyst	12
Reasons of further therapy	
Appearance of mural nodule	6
MPD ≥10 mm	1
Appearance of mural nodule + MPD ≥10 mm	2
Cyst Diameter ≥30 mm plus patient’s proposal	2
Penetration to stomach plus cyst Diameter ≥30 mm	1
Modalities for diagnosis	
Surgical specimen	9
Pancreatic juice cytology plus imaging examinations	3
Diagnosis	
IPMN with LGD	5
IPMN with HGD	3
Equivalent to IPMN with HGD^a^ MPD ≥10 mm, cytologically positive for HGD, not resected case Mural nodule positive, cytologically positive for LGD, not resected case	2
IPMN associated with invasive carcinoma (stage 3, UICC 8^th^ ed.)^a^ (not resected case)	1
SCN	1
Duration time from initial diagnosis to onset of IPMN with HGD or associated invasive carcinoma, median (range), months	85 (24 - 174)
Concomitant PDAC	14
Stage 0, 1, 2a, 2b, 3, 4	0,0,8,4,0,2
Modalities for diagnosis	
Surgical specimen	12
EUS-FNA plus imaging examinations	2
Duration time from initial diagnosis to onset of concomitant PDAC, median (range), months	45 (14 - 119)

*MPD* main pancreatic duct, *IPMN* intraductal papillary mucinous neoplasm, *LGD* low-grade dysplasia, *HGD* high-grade dysplasia, *PDAC* pancreatic ductal adenocarcinoma, *SCN* serous cystic neoplasm, *EUS-FNA* fine needle aspiration using endoscopic ultrasonography

^a^The patients were diagnosed with pancreatic juice cytology using endoscopic retrograde pancreatography plus imaging studies

### Risk factors for concomitant pancreatic cancer with pancreatic cyst

We studied the risk factors for concomitant PDAC in all 547 patients (Table 3). All 14 concomitant PDACs came from patients with IPMNs, and there were no concomitant PDACs in the 99 patients with Others. There were significant differences for dilated MPD (≥2.5 mm) and diabetes mellitus in univariate analysis, but diabetes mellitus remained the sole risk factor for concomitant PDAC in multivariate analysis. Next, we analyzed the risk factors for concomitant PDAC, focusing on patients with IPMNs (Table 4). Diabetes mellitus on initial diagnosis was a distinct item that was related to concomitant PDAC, as determined in both the univariate and multivariate analyses. Furthermore, the carcinogenesis in IPMN plus diabetes mellitus was at frequency of 9.5% [9/95] at the median 48-month observation.

**Table 3:** Risk Factors for concomitant PDAC with all of 547 patients with pancreatic cyst

	Concomitant PDAC (*N* = 14)	Exception of Concomitant PDAC (*N* = 533)	Univariate Analysis (*P*-value)	Multivariate Analysis	
				Odds ratio (95% CI)	(*P*-value)
Follow-up Period, median (range), months	45 (14-119)	59 (13-177)	0.30		
Sex, M:F	7:7	197:336	0.4		
Age at Cyst Diagnosis, median (range), years	73 (56-80)	71 (31-93)	0.79		
Age at Cyst Diagnosis ≥65 years old	12:2	385:149	0.37	1.98 (0.24-16.29)	0.52
Age at Final Observation, median (range), years	76.5 (60-86)	76 (32-99)	0.79		
Age at Final Observation ≥70 years old	12:2	391:142	0.37		
Cyst Number (1,2, ≥3)	2:4:8	222:118:193			
Cyst Number (1, ≥2)	2:12	222:312	0.05	0.37 (0.05-2.43)	0.30
Cyst Diameter, median (range), mm	17.0 (6.4-27.6)	14.5 (2-68)	0.17		
Cyst Diameter (mm) ≥14.6, <14.6	10:14	263:270	0.11	2.38 (0.43-13.27)	0.32
Diameter of MPD, median (range), mm	3.1 (1-7.7)	2.5 (1-9.8)	0.06		
MPD (mm) ≥2.5, <2.5	11:3	192:213	0.03	2.88 (0.45-18.27)	0.26
IPMN: non-IPMN	14:0	425:108	0.08		
Diabetes Mellitus at Cyst Diagnosis, yes : no	9:5	103:430	<0.01	304.58 (51.01-1818.74)	<0.001
Hypertension at Cyst Diagnosis, yes : no	9:5	237:296	0.18		
Hyperlipidemia at Cyst Diagnosis, yes : no	7:7	138:395	0.06	3.61 (0.77-16.88)	0.10
Cancer History, yes : no	2:12	150:537	0.75		
Usual Alcohol Consumption ethanol (ethanol ≥20g/day	5:9	156:378	0.56		
Smoking (BI ≥400), yes : no	3:11	135:398	1.00		
Family History of Pancreatic Cancer (≤1^nd^ grade), yes : no	1:12	39:491	1.00		
BMI ≥25(kg/m^2^), yes : no	1:13	114:419	0.32		

*PDAC* Pancreatic ductal adenocarcinoma, *CI *Confidence interval*, M* male, *F* female, *MPD* main pancreatic duct, *IPMN* intraductal papillary mucinous neoplasm, *HT* hypertension, *HL* hyperlipidemia, *BMI* body mass index

14.6 mm in cyst diameter and 2.5mm in MPD diameter were overall median

There were 4 data deficiencies in family history of pancreatic cancer

**Table 4:** Risk factors for concomitant PDAC with 439 Patients with IPMN

	Concomitant PDAC (*N* = 14)	Exception of Concomitant PDAC (*N* = 425)	Univariate Analysis (*P* - value)	Multivariate Analysis	
				Odds ratio (95% CI)	*P*-value
Follow-up Period, median (range), months	44.5 (14-119)	57 (13-177)	0.24		
Sex, M:F	7:7	162:263	0.41		
Age at Cyst Diagnosis, median (range), years	73 (56-80)	71 (35-93)	1.00		
Age at Cyst Diagnosis ≥ 65 years old	12:2	307:118	0.37	1.41 (0.29-6.88)	0.67
Age at Final Observation, median (range), years	76.5 (60-86)	77 (39-99)	1.00		
Age at Final Observation ≥70 years old	12:2	324:101	0.6		
Cyst Number (1,2, ≥3)	2:4:8	139:105:181			
Cyst Number (1, ≥2)	2:12	139:286	0.24		
Cyst Diameter, median (range), mm	17.0 (6.4-27.6)	14.9 (5-56.3)	0.17		
Cyst Diameter (mm) ≥15.2, <15.2	10:4	209:216	0.11	1.80 (0.54-6.03)	0.34
Diameter of MPD, median (range), mm	3.1 (1-7.7)	2.5 (1-9.8)	0.10		
MPD (mm) ≥2.5, <2.5	11:3	230:197	0.10	2.57 (0.67-9.87)	0.17
Diabetes Mellitus at Cyst Diagnosis, yes : no	9:5	86:339	<0.001	5.40 (1.69-17.22)	<0.01
Hypertension at Cyst Diagnosis, yes : no	9:5	189:236	0.18		
Hyperlipidemia at Cyst Diagnosis, yes : no	7:7	105:320	0.05	2.02 (0.66-6.24)	0.22
Cancer History, yes : no	2:12	117:308	0.37		
Usual Alcohol Consumption (ethanol ≥20g/day)	5:9	124:304	0.56		
Smoking (BI ≥400), yes : no	3:11	116:309	0.77		
Family History of Pancreatic Cancer ( ≤ 1^nd^ grade ), yes : no	1:12	31:392	1.00		
BMI (kg/m2) ≥25, yes : no	1:13	86:339	0.32		

*PDAC* Pancreatic ductal adenocarcinoma, *CI* Confidence interval, *M* male, *F* female, *MPD* main pancreatic duct, *IPMN* intraductal papillary mucinous neoplasm, *BMI* body mass index

15.2mm in cyst diameter and 2.5mm in MPD diameter were overall median

There were 4 data deficiencies in family history of pancreatic cancer

### Analysis of the incidence rate of concomitant PDAC using the Kaplan–Meier method

**Fig. 3:**
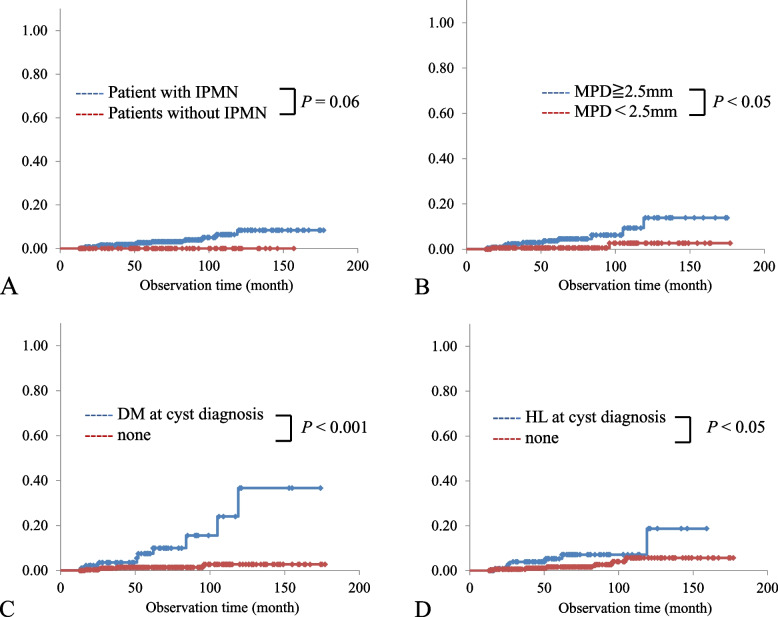
Analysis of risk factors for concomitant PDAC with Kaplan–Meier Method in our 547patient cohort overall and in 439 patients with IPMN. In analysis of all patients, between patients with IPMN and nonIPMN, there was no statistically significant differences (*P* = 0.06) (**a**). In analysis focusing on 439 patients with IPMN, MPD dilatation (≥2.5 mm; *P* < 0.05) (**b**), diabetes mellitus (*P* < 0.001) **(c)**, and hyperlipidemia (*P* < 0.05) (**d**) at initial cyst diagnosis concerned with higher incidence rate of concomitant PDAC in univariate analysis. IPMN, intraductal papillary mucinous neoplasm; MPD, main pancreatic duct; DM, diabetes mellitus; HL, hyperlipidemia

We evaluated the incidence rate of concomitant PDAC in all patients and in patients with IPMNs using the Kaplan–Meier method. Supplementary Tables 3 and 4 show a comparison of the incidence rates at 5 years and 10 years for various parameters. Representative cases are shown in Figs. 3a, b, c, and d. In all 549 patients (Supplementary Table 3), diabetes mellitus (*P* < 0.001), hyperlipidemia (*P* < 0.05), dilated MPD (≥2.5 mm) (*P* < 0.05), and multiple cysts (*P* < 0.05) demonstrated a significantly higher risk in the univariate analysis, but diabetes mellitus was the only item in the multivariate analysis (*P* <0.01). Next, when focusing on IPMN (Supplementary Table 4), diabetes mellitus (*P* < 0.001), hyperlipidemia (*P* < 0.05), and dilated MPD (≥2.5 mm) (*P* < 0.05) demonstrated significantly higher risks in the univariate analysis, but diabetes mellitus was the only item in the multivariate analysis (*P* < 0.001).

### Standardized incidence ratio of pancreatic carcinoma

**Fig. 4:**
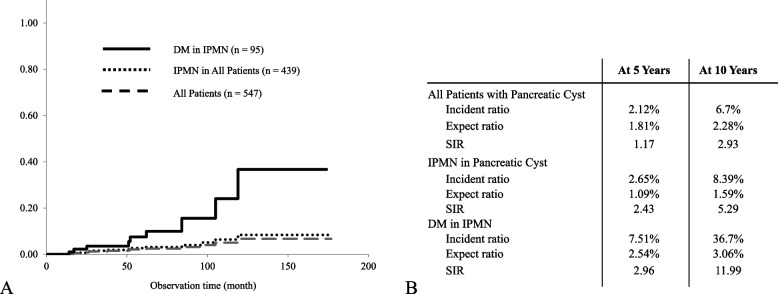
(**a**) Cumulative carcinogenic curve in all patients with cyst, patients with intraductal papillary mucinous neoplasm (IPMN), and IPMN plus diabetes mellitus (DM). (**b**) Cumulative carcinogenic ratio (Incident ratio), Expected ratio in the general Japanese population (Expect ratio), and Standardized incidence ratio (SIR) in each group

We calculated the expected incidence ratio of pancreatic cancer in the general Japanese population for each group. The rations for all patients, patients with IPMNs, and patients with IPMNs plus diabetes mellitus were 1.81%, 1.09%, and 2.54% at 5 years and 2.28%, 1.59%, and 3.06% at 10 years, respectively. The cumulative carcinogenic rate within each group was calculated with the Kaplan–Meier method, as shown in Supplementary Tables 3 and 4 and Fig. 4a. Using these results, the SIRs for concomitant PDAC of all patients, patients with IPMNs, and patients with IPMN plus diabetes mellitus were 1.17, 2.43, and 2.96 at 5 years and 2.93, 5.29, and 11.99 at 10 years, respectively (Fig. 4b).

### Status of diabetes mellitus in patients with IPMN-derived carcinomas and concomitant PDACs

In 5 IPMNs with HGD and one IPMN associated with invasive carcinoma, there was a frequency of coexistence with LSDM (4/6, 66.7%) (Supplementary Table 1). All 9 patients with diabetes mellitus and concomitant PDAC did not have new-onset diabetes mellitus (NODM) (duration <2 years) but rather long-standing diabetes mellitus (LSDM) (duration ≥2 years). The median period from onset of diabetes mellitus to induction of concomitant PDAC was 12 years (median, 2.5–22) (Supplementary Table 5).

## Discussion

A few reports have indicated risk factors for concomitant PDAC in patients with IPMN; thus, we need to follow all patients with IPMN in a uniform manner with consideration of the possible onset of concomitant PDAC. This study is the first to report that long-standing diabetes mellitus is a risk factor for concomitant PDAC in patients with IPMN, and this might be an indication to reconsider the surveillance method for IPMNs.

Pancreatic cancers that developed from IPMN are divided into the two following types: (1) carcinogenesis from IPMN itself (IPMN-derived carcinoma) and (2) carcinoma development away from IPMN (concomitant PDAC). However, there are no guidelines and recommendations for surveillance that are concerned with finding concomitant PDAC [4–7]. Herein, we encountered 14 patients with concomitant PDAC in pancreatic cysts, especially in IPMNs, and analyzed the risk factors for concomitant PDAC.

First, the proportion of the incident rate (IPMN-derived carcinoma vs. concomitant PDAC) reportedly varies (4:1, 1:1, 2:5) [10, 11, 14]. In our cohort, we encountered only 5 IPMNs with high-grade dysplasia and one IPMN associated with invasive carcinoma. In contrast, 14 patients had concomitant PDACs. Our accommodation for surgery was not based on cyst size. One patient with a cyst of 68 mm in size had penetration into the stomach and underwent surgery, but the histological diagnosis was IPMN with HGD. As such, in our cohort, there might be some patients who did not have an operation unless they had a larger cyst, possibly with high-grade dysplasia. However, there have been no critical problems for these patients until now. Furthermore, most patients with further therapies due to worsening of a cyst had a better prognosis than those with concomitant PDAC. From this result and other studies [10, 11], we strongly recognize the necessity of surveillance with targets for concomitant PDAC in a patient with IPMNs.

The mechanism underlying the frequent occurrence of concomitant PDAC in patients with IPMNs is not yet fully understood. The most likely explanation is that patients with IPMNs often have concurrent pancreas intraepitherial neoplasia (PanIN) or small gastric-type IPMN lesions that develop into PDAC [15, 16]. For these reasons, most physicians in Japan might perform surveillance only for IPMNs diagnosed with international guidelines. In contrast, we performed surveillance for not only IPMNs but also all cystic lesions. The reasons for our surveillance of patients with all cysts are based on the following two concepts: (1) the connection between cysts and MPD is not completely distinguishable with the use of any of the modalities we used; and (2) cysts diagnosed as non-IPMN are mostly small and round and classified as simple cysts or retention cysts. These two cyst types are difficult to distinguish by imaging examinations. PanINs can be a cause of retention cysts. For these reasons, we continued surveillance for all cysts twice a year until a patient’s physical status indicated difficulties for surgery.

Currently, we are reconsidering whether such strict adherence to surveillance for all patients with cysts is proper. We are reconsidering whether it may impose an undue demand on patients and doctors and affect health economics. In our cohort, only 14 out of 547 patients with a cyst developed concomitant PDACs in a 14-year period (2.6%). This represents only 2.8% (14/495) of all patients with PDAC at our institute between April 2007 and June 2020 (detailed data not shown). Furthermore, pancreatic cysts are more frequently being detected, with a reported prevalence of 2.1-2.6% using CT [6] and 13.5-45% using MRI/MRCP [6]. The surveillance of all cysts might not be cost-effective and may impose an undue burden on health care workers and affect medical economics. Thus, we should apply a surveillance method according to the carcinogenic risk of each person.

First, we analyzed whether IPMNs diagnosed using international guidelines more often had concomitant PDAC than non-IPMNs. All 14 patients with concomitant PDAC had IPMNs, and all 99 patients with Others had no concomitant carcinoma. The carcinogenic rate at 10 years was 2.28% (SIR: 2.93) in the overall cohort and 8.39% (SIR: 5.29) in patients with IPMNs. Thus, IPMNs seem to have more concomitant PDACs than other cysts.

Next, we analyzed risk factors for concomitant PDAC in patients with IPMNs and found that diabetes mellitus, especially LSDM, was a strong risk factor for concomitant PDAC. Diabetes mellitus in patients with IPMNs had a risk of 9.5% (9/95) (median 48-month follow-up period), and the 10-year incidence rate and SIR were 36.7% and 11.99, respectively. In addition, the carcinogenic rate increased fourfold at 10 years compared with that at 5 years. Thus, the risk of concomitant PDAC might increase over time.

There are few reports on the risk factors for concomitant PDAC in patients with IPMNs. Uehara mentioned that patients over 70 years of age had a 19.4-fold increased risk of concomitant PDAC [11]. In our study, the median age of concomitant PDAC was 77 years, with 12 of 14 PDAC patients being above 70 years old. There was no statistically significant difference detected in this cohort, but high age must be a strong risk factor for PDAC. Nehra [17] and Mandai [18] reported that the FH of PDAC increased the risk of concomitant PDAC in patients with IPMNs. The risks were high (11.1% for the FH of second degree and 17.6% for the FH of first degree), and Maindai reported that the risk normalized in patients aged ≥70 years old. As FH is a well-known and salient risk factor for PDAC, it is also necessary to pay adequate attention to the FH of PDAC, especially in patients aged < 70 years old. Unlike our results, Pergolini [14] reported that concomitant PDACs were not associated with diabetes mellitus.

In typical PDACs, the risk factors are well known; they include the FH of PDAC, hereditary pancreatic cancer syndrome, IPMN [4–8], smoking, chronic pancreatitis, obesity, and diabetes mellitus. The association between diabetes mellitus and the risk of PDACs has been evidenced in numerous studies, and diabetes mellitus has been reported to carry a higher risk for PDACs (1.8–2.5-fold) [19–23]. In particular, NODM has a very high risk for PDACs (2.9–6.56-fold) [17–21]. These changes are considered to stem from the destruction of the pancreas or the paraneoplastic effects of PDACs. Thus, NODM is a very important risk factor for finding PDACs [24]. Moreover, the risk of PDACs in LSDM is relatively lower (1.5–2.5-fold) [19, 21, 23, 25] than that in NODM.

LSDM (especially type 2) is considered to increase carcinogenic factors via high insulin resistance and hyperglycemia. Hyperinsulinemia from increased insulin resistance might upregulate cell growth, downregulate apoptosis and facilitate carcinoma formation [26]. Hyperglycemia induces excessive oxidative stress via overoxidation of the mitochondria [27] and induces DNA damage [28]. Interestingly, in this study, hyperlipidemia and hypertension also tended to be related to the onset of concomitant carcinomas. Both of these factors stem from insulin resistance and increased oxidative stress and might be a factor in carcinogenesis. Furthermore, there are some reports that diabetes mellitus promotes the onset and carcinogenesis of IPMNs [29, 30]. Concomitant PDAC is thought to result from PanIN or small gastric-type IPMNs away from a cyst, and LSDM might work as a promoter of PDAC.

We made some presumptions regarding the ideal surveillance method for IPMN being inappropriate for surgery on initial diagnosis. First, routine surveillance should be performed according to each guideline and should be mainly concerned with cyst status during the initial five years. In addition, a new scoring model [31] for the prediagnosis of malignancies in patients with IPMN has been reported that we could utilize for diagnosing IPMN-derived carcinomas. Next, surveillance should be concerned with possible concomitant PDAC. For this purpose, it might be desirable to perform the continuous surveillance of all IPMNs twice a year. However, given the relatively low frequency of concomitant PDAC in patients with IPMN, we could set the examination frequency according to whether patients had other risk factors. Initially, we could concentrate on IPMNs per the 2017 international guidelines and not SCNs and Others. This is because our results indicated that all concomitant PDACs originated from IPMNs and not SCNs and Others. Hard surveillance with multiple modalities twice a year might be effective for elderly people with IPMNs plus diabetes mellitus. In contrast, mild surveillance with a simple modality once a year might be sufficient for young patients with solely IPMNs. We need more findings regarding risk factors for concomitant PDAC in patients with IPMNs.

This study had several limitations. First, this was a retrospective cohort, although data were prospectively collected. As such, there were some data deficiencies, such as incomplete family histories for PDAC. Next, there might have been a hospital bias. Most cystic lesions are only discovered incidentally on imaging examinations, so most patients in our cohort had other diseases or suspicion of other diseases. Accordingly, there were many patients with other diseases, including malignancies. Furthermore, there were more females than males in our cohort because our hospital had many patients with breast and uterine diseases.

In conclusion, during the surveillance of IPMN cases inappropriate for surgery on initial diagnosis, it is important to pay attention to the possible development of concomitant PDACs. However, the incident rate is relatively low, so surveillance plans for each patient should consider other risk factors for PDAC, especially older age, a FH of PDAC, and LSDM. In addition, it might be more effective to concentrate only on high-risk patients with IPMNs, and the remaining resources should be targeted toward medical checkups for the general population without risk factors.

## Supplementary Information

**Additional file 1.**

**Additional file 2.**

**Additional file 3.**

**Additional file 4.**

**Additional file 5.**

## Data Availability

The datasets used and/or analysed during the current study are available from the corresponding author on reasonable request.

## Abbreviations

PDAC: Pancreatic ductal adenocarcinoma
IPMN: Intraductal papillary mucinous neoplasm
MPD: Main pancreatic duct
FH: Family history
CE-CT: Abdominal contrast enhanced computed tomography scans
MRCP: Magnetic resonance cholangiopancreatography
EUS: Endoscopic ultrasonography
SCN: Serous cystic neoplasm
HGD: High-grade dysplasia
SIR: Standardized incident ratio
LGD: Low-grade dysplasia
NODM: New-onset diabetes mellitus
LSDM: Long-standing diabetes mellitus
