# Supplementary material for: Long-standing diabetes mellitus increases concomitant pancreatic cancer risk in patients with intraductal papillary mucinous neoplasms
Source: BMC Gastroenterol. 2022 Dec 20;22:529. doi: 10.1186/s12876-022-02564-8 (PMC9764692; doi:10.1186/s12876-022-02564-8)
Supplement: Supplementary file 1 — Additional file 1. [file 12876_2022_2564_MOESM1_ESM.docx]

SUPPLEMENTARY TABLE 1. Characteristics of Patients who had Further Therapy due to Worsening of Cyst

|  | Age | M/F | The Reasons For Further Therapy | Further Therapy | Diagnosis | Characteristics of Cyst at Initial Diagnosis | | | | |
| --- | --- | --- | --- | --- | --- | --- | --- | --- | --- | --- |
|  |  |  |  |  |  | Cyst Type | Max. Cyst Diameter (mm) | Numbers of cyst  (1,2, ≥3) | Diameter of MPD (mm) | Mural Nodule |
| 1 | 83 | M | Appearance of nodule  MPD ≥10mm | Operation | IPMN (HGD) | IPMN | 9.8 | 1 | 3 | None |
| 2 | 69 | M | Appearance of nodule | Operation | IPMN (LGD) | IPMN + Others | 30.2 | 2 | 1.5 | None |
| 3 | 86 | M | MPD ≥10mm | Radiation | s/o IPMN (HGD) * | IPMN | 13.2 | 1 | 7 | None |
| 4 | 64 | M | Patient's request, Cyst ≥30mm | Operation | IPMN (LGD) | Others | 32.1 | 1 | 1.6 | None |
| 5 | 64 | F | Appearance of nodule | Operation | IPMN (LGD) | IPMN | 29.5 | 1 | 1.9 | None |
| 6 | 82 | M | Penetration to stomach | Operation | IPMN (HGD) | Others | 68 | 2 | 1.5 | None |
| 7 | 85 | F | Appearance of nodule | Operation | IPMN (LGD) | IPMN | 23.8 | >3 | 4.3 | None |
| 8 | 62 | M | Appearance of nodule | Operation | IPMN (LGD) | IPMN | 31.3 | 2 | 3.4 | None |
| 9 | 40 | F | Patient's request, Cyst ≥30mm | Operation | SCN | IPMN | 36.1 | 1 | 1 | None |
| 10 | 75 | M | Appearance of nodule, MPD ≥10mm | Operation | IPMN (HGD) | IPMN | 11.8 | >3 | 3.8 | None |
| 11 | 82 | F | PDAC derived from IPMN | Chemotherapy | PDAC derived from IPMN (Stage3) | IPMN | 11.8 | 3> | 3.8 | None |
| 12 | 86 | M | Appearance of nodule | Observation | s/o IPMN (HGD) * | IPMN | 35.1 | 3> | 2.9 | None |

M=male, F=female, MPD=main pancreatic duct, PDAC=pancreatic ductal adenocarcinoma, IPMN=intraductal papillary mucinous neoplasm, HGD=high-grade dysplasia, LGD=low-grade dysplasia, s/o=suspect of, SCN=serous cystic neoplasm

Others is defined as non-IPMN/SCN cyst.

* Patients (number 3 and 12) were diagnosed with pancreatic juice cytology plus imaging studies.

SUPPLEMENTARY TABLE 1. Continue

|  | Age at initial diagnosis (years old) | Observation time to event (month) | Characteristics of Patients at Initial Diagnosis | | | | | | Prognosis |
| --- | --- | --- | --- | --- | --- | --- | --- | --- | --- |
|  |  |  | Diabetes Mellitus | History of Malignancy | Usual Alcohol Intake | Smoking (BI ≥ 400) | Family History of　Pancreatic Cancer (　≤　2^nd^ degree) | BMI ≥ 25 (kg/mm^2^) |  |
| 1 | 69 | 174 | ● | × | ● | ● | × | × | Arrive in 22 month with no relapse |
| 2 | 56 | 155 | ● | × | ● | ● | ● | ● | Death associated with operation |
| 3 | 78 | 99 | × | Hepatocellular Cancer | × | ● | × | ● | Death in 84.7 month from another reason with no relapse |
| 4 | 52 | 104 | ● | Gallbladder Cancer | ● | ● | × | × | Arrive in 38.5 month with no relapse |
| 5 | 58 | 40 | × | × | × | × | × | ● | Arrive in 110.9 month with no relapse |
| 6 | 75 | 85 | × | Rectal Cancer | ● | × | × | ● | Arrive in 49.7month with no relapse |
| 7 | 81 | 41 | ● | × | × | × | ● | ● | Arrive in 72.9 month with no relapse |
| 8 | 60 | 26 | ● | × | ● | ● | ● | × | Arrive in 74 month with no relapse |
| 9 | 38 | 24 | × | × | × | × | × | × | Arrive in 64.5 month with no relapse |
| 10 | 70 | 58 | ● | × | ● | ● | × | ● | Death in 23.9 month from another reason with no relapse |
| 11 | 77 | 66 | ● | Endometrial Cancer | × | × | ● | × | Transfer to another hospital in 2.9 month |
| 12 | 84 | 24 | ● | Prostate Cancer | × | × | × | ● | Arrive in 5.6 month without therapy |

BI=brinkman Index, BMI=Body Mass Index, ●=yes, ×= none
