# Supplementary material for: Long-standing diabetes mellitus increases concomitant pancreatic cancer risk in patients with intraductal papillary mucinous neoplasms
Source: BMC Gastroenterol. 2022 Dec 20;22:529. doi: 10.1186/s12876-022-02564-8 (PMC9764692; doi:10.1186/s12876-022-02564-8)
Supplement: Supplementary file 2 — Additional file 2. [file 12876_2022_2564_MOESM2_ESM.docx]

|  | Age | M/F | Obser-vation interval | Modality for surveillance | Reason for notification of PDAC | Location of PDAC | Further Therapy | Tumor Size  (mm) | Stage | Characteristics of Cyst at Initial Diagnosis | | | | | |
| --- | --- | --- | --- | --- | --- | --- | --- | --- | --- | --- | --- | --- | --- | --- | --- |
|  |  |  |  |  |  |  |  |  |  | Cyst Type | Max. Cyst Diameter (mm) | Numbers of Cyst  (1,2, ≥3) | Location of Cyst | Diameter of MPD (mm) | Mural Nodule |
| 1 | 60 | F | 6M | CE-CT | CE-CT | Tail | Operation | 19 | 2a | IPMN | 27.5 | 1 | H | 1 | None |
| 2 | 85 | M | 12M | MRCP | Jaundice | Head | No therapy | 19 | 4* | IPMN + Others | 15.6 | 2 | H, B | 3.2 | None |
| 3 | 86 | M | 6M | MRCP | MRCP | Tail | Operation | 28 | 2b | IPMN | 25.7 | ≥3 | U,H,T | 3.5 | None |
| 4 | 83 | F | 12M | CT | CT | Tail | No therapy | 40 | 4* | IPMN | 20.5 | 2 | H, T | 4 | None |
| 5 | 77 | M | 12M | MRCP | MRCP | Head | Operation | 32 | 2b | IPMN + Others | 16.9 | ≥3 | U,H,B,T | 3.4 | None |
| 6 | 73 | F | 6M | MRCP/CE-CT | CE-CT | Head | Operation | 25 | 2b | IPMN | 17 | 2 | H | 2.9 | None |
| 7 | 72 | M | 6M | MRCP/CE-CT | MRCP | Head | Operation | 25 | 2a | IPMN | 10.2 | ≥3 | H, B, T | 2.3 | None |
| 8 | 71 | M | 6M | MRCP/CE-CT | MRCP | Tail | Operation | 15 | 2a | IPMN | 14.2 | 2 | B,T | 3.5 | None |
| 9 | 74 | F | 6M | MRCP/CE-CT | CE-CT | Head | Operation | 18 | 2a | IPMN | 20 | ≥3 | U, B | 3.3 | None |
| 10 | 81 | F | 6M | MRCP | MRCP | Tail | Operation | 17 | 2a | IPMN | 15.5 | ≥3 | U, B,T | 2 | None |
| 11 | 84 | F | 6M | MRCP/CE-CT | MRCP | Head | Operation | 32 | 2a | IPMN + Others | 12.7 | ≥3 | U, H, B,T | 2.5 | None |
| 12 | 76 | F | 6M | MRCP/CE-CT | MRCP | Tail | Operation | 14 | 2a | IPMN + Others | 6.4 | ≥3 | U, B | 2.7 | None |
| 13 | 62 | M | 6M | MRCP/CE-CT | Obstruction of duodenum | Groove | Operation | 60 | 2b | IPMN | 24.9 | 1 | H | 2.6 | None |
| 14 | 78 | M | 12M | MRCP | MRCP | Body | Operation | 17 | 2a | IPMN | 27.6 | ≥3 | H, B,T | 7.7 | None |

SUPPLEMENTARY TABLE 2. Concomitant PDACs in all of 547 Patients with Cyst

M=male, F=female, 6M=every 6 months, 12M=every 12 months, PDAC=pancreatic ductal adenocarcinoma, CT=computed tomography, CE-CT=contrast-enhaced computed tomography, MRCP=magnetic resonance cholangiopancreatography, IPMN=intraductal papillary mucinous neoplasm, U=uncinate process of the pancreas, H=head of the pancreas, B=body of the pancreas, T=tail of the pancreas, MPD=main pancreatic duct

Stages of tumor are presented using with 8^th^ edition of UICC TNM classification.

Others are defined as non-IPMN or non- SCN cyst.

* Patients (number 2 and 4) were diagnosed with fine needle aspiration using endoscopic ultrasonography plus imaging studies.

SUPPLEMENTARY TABLE 2 .　Continue

|  | Age at initial diagnosis (years old) | Observation time to event (month) | Characteristics of patients at initial diagnosis | | | | | | Prognosis |
| --- | --- | --- | --- | --- | --- | --- | --- | --- | --- |
|  |  |  | Diabetes Mellitus | History of malignancy | Usual alcohol intake | Smoking (BI ≥400) | Family history of pancreatic cancer ( ≤ 2^nd^ degree) | BMI≥25 (kg/mm2) |  |
| 1 | 56 | 51 | ● | Rectal carcinoma | × | × | × | ● | Transfer to another hospital after 73.1 month with no relapse |
| 2 | 80 | 52 | ● | × | × | × | unknown | × | Death in 3.2 month with PC |
| 3 | 76 | 119 | ● | Gastric cancer | × | × | × | × | Death in 5.4 month at another disease with no relapse |
| 4 | 74 | 84 | ● | × | × | × | × | × | Transfer to another hospital after 2.1 month with no relapse |
| 5 | 68 | 105 | ● | × | ● | ● | × | × | Death in 19.8 month with PC |
| 6 | 70 | 38 | × | × | × | × | ● | × | Death in 7.8 month with PC |
| 7 | 65 | 96 | × | × | × | ● | × | × | Arrive in 9.8 month with no relapse |
| 8 | 69 | 25 | ● | × | ● | × | × | × | Death in 17.3 month with PC |
| 9 | 72 | 27 | × | × | ● | × | × | × | Death in 50.5 month with PC |
| 10 | 80 | 17 | ● | × | × | × | × | × | Death in 66.5 month at another disease with no relapse |
| 11 | 79 | 62 | ● | × | × | × | × | × | Arrive in 12.1 month with no relapse |
| 12 | 75 | 17 | × | × | × | × | × | × | Arrive in 58.8 month with no relapse |
| 13 | 60 | 26 | × | × | ● | ● | × | × | Arrive in 14.1 month with relapse at live, lung, and peritoneum |
| 14 | 77 | 14 | ● | × | ● | × | × | × | Transfer to another hospital in 3.7 month with no relapse |

BI=Brinkman index, BMI=body mass index, ●=yes, ×= none, PC=Pancreatic cancer
