# Supplementary material for: Long-standing diabetes mellitus increases concomitant pancreatic cancer risk in patients with intraductal papillary mucinous neoplasms
Source: BMC Gastroenterol. 2022 Dec 20;22:529. doi: 10.1186/s12876-022-02564-8 (PMC9764692; doi:10.1186/s12876-022-02564-8)
Supplement: Supplementary file 3 — Additional file 3. [file 12876_2022_2564_MOESM3_ESM.docx]

|  | Cumulative Incident Ratio (%) | | | | Univariate Analysis | Multivariate Analysis | |
| --- | --- | --- | --- | --- | --- | --- | --- |
|  | Present | | None | | *P* - value | HR (95% CI) | *P* - value |
|  | 5-year | 10-year | 5-year | 10-year |  |  |  |
| Age at Cyst Diagnosis ≥65 | 2.3 | 10.6 | 1.6 | 1.6 | 0.09 | 1.66 (0.34-8.17) | 0.54 |
| Cyst Number ≥2 | 2.8 | 11.1 | 1.2 | 1.2 | ＜0.05 | 0.31 (0.07-1.46) | 0.14 |
| Cyst Diameter ≥14.6 mm | 1.3 | 10.5 | 0.8 | 3.2 | 0.08 | 1.76 (0.51-6.04) | 0.37 |
| MPD Diameter ≥2.5mm | 3.7 | 12.0 | 1.1 | 2.7 | 0.04 | 2.23 (0.58-8.55) | 0.24 |
| Cysts with IPMN | 2.7 | 8.4 | 0 | 0 | 0.06 |  |  |
| Diabetes Mellitus at Cyst Diagnosis | 6.4 | 32.5 | 1.1 | 2.1 | ＜0.001 | 6.56 (2.00-21.49) | ＜0.01 |
| Hypertension at Cyst Diagnosis | 3.3 | 5.8 | 7.2 | 3.2 | 0.06 | 1.19 (0.35-4.08) | 0.78 |
| Hyperlipidemia at Cyst Diagnosis | 4.2 | 13.3 | 1.3 | 4.7 | ＜0.05 | 1.67 (0.54-5.22) | 0.37 |

SUPPLEMENTARY TABLE 3. Cumulative Carcinogenic Risk and Risk Factors for Pancreatic Cancer in all of 547 Patients

HR=Hazard ratio, MPD=Main pancreatic duct, IPMN=Intraductal papillary mucinous neoplasm

Univariate analysis and multivariate analysis was performed with log-rank test with Kaplan-Meier method and cox proportional hazard model, respectively.
