# Supplementary material for: Long-standing diabetes mellitus increases concomitant pancreatic cancer risk in patients with intraductal papillary mucinous neoplasms
Source: BMC Gastroenterol. 2022 Dec 20;22:529. doi: 10.1186/s12876-022-02564-8 (PMC9764692; doi:10.1186/s12876-022-02564-8)
Supplement: Supplementary file 4 — Additional file 4. [file 12876_2022_2564_MOESM4_ESM.docx]

|  | Cumulative Incident Ratio (%) | | | | Univariate Analysis | Multivariate Analysis | |
| --- | --- | --- | --- | --- | --- | --- | --- |
|  | Present | | None | | *P* - value | HR (95% CI) | *P* - value |
|  | 5-year | 10-year | 5-year | 10-year |  |  |  |
| Age at cyst diagnosis ≥65 | 2.8 | 12.7 | 2.7 | 2.7 | 0.14 | 2.75 (0.29-25.8) | 0.38 |
| Cyst Number ≥2 | 3.0 | 1.9 | 12.5 | 1.9 | 0.09 | 3.10 (0.36-26.82) | 0.30 |
| Cyst diameter ≥15.2 mm | 4.5 | 14.3 | 1.0 | 3.9 | 0.06 | 2.80 (0.53-14.71) | 0.22 |
| MPD diameter ≥2.5mm | 3.7 | 13.9 | 1.4 | 3.5 | ＜0.05 | 1.06 (0.24-4.58) | 0.94 |
| BMI ≥25 mm^2^ / kg | 2.3 | 2.3 | 2.8 | 9.7 | 0.27 |  |  |
| Usual alcohol drinking | 2.6 | 10.0 | 2.6 | 7.4 | 0.65 |  |  |
| Smoking (BI ≥400) | 1.0 | 9.7 | 3.3 | 7.6 | 0.63 |  |  |
| History of malignancies | 1.5 | 1.6 | 3.1 | 7.9 | 0.36 |  |  |
| Diabetes mellitus at cyst diagnosis | 7.5 | 36.7 | 1.4 | 2.7 | ＜0.001 | 7.69 (1.77-33.45) | ＜0.01 |
| Hypertension at cyst diagnosis | 4.1 | 16.1 | 1.6 | 4.6 | 0.06 | 2.55 (.46-14.00) | 0.28 |
| Hyperlipidemia at cyst diagnosis | 5.3 | 18.7 | 1.7 | 5.7 | ＜0.05 | 1.38 (0.36-5.29) | 0.64 |
| Family history of pancreatic cancer ≤ 2^nd^ degree | 3.9 | 3.9 | 2.1 | 8.4 | 0.98 |  |  |

SUPPLEMENTARY TABLE 4. Cumulative Carcinogenic Risk and Risk Factors for Pancreatic Cancer in Patients with IPMN

HR=Hazard ratio, MPD=Main pancreatic duct, BMI=Body mass index, BI=Brinkman index

Univariate analysis and multivariate analysis was performed with log-rank test with Kaplan-meier method and cox proportional hazard model, respectively.
