# Supplementary material for: Long-standing diabetes mellitus increases concomitant pancreatic cancer risk in patients with intraductal papillary mucinous neoplasms
Source: BMC Gastroenterol. 2022 Dec 20;22:529. doi: 10.1186/s12876-022-02564-8 (PMC9764692; doi:10.1186/s12876-022-02564-8)
Supplement: Supplementary file 5 — Additional file 5. [file 12876_2022_2564_MOESM5_ESM.docx]

SUPPLEMENTARY TABLE 5. Condition of Diabetes Mellitus in 14 Cases Developed in Concomitant Pancreatic Ductal Adenocarcinoma

| Case | M/F | Age at Pancreatic Cancer (years old) | Age at Cyst Diagnose (years old) | Diabetes Mellitus at Cyst Diagnosis | Age at Onset of Diabetes Mellitus (years old) | Time from Diabetes Mellitus to Pancreatic Cancer (years) |
| --- | --- | --- | --- | --- | --- | --- |
| 1 | F | 60 | 56 | ● | 40 | 20 |
| 2 | M | 85 | 80 | ● | Before 78 | Longer than 7 |
| 3 | M | 86 | 76 | ● | 63 | 23 |
| 4 | F | 83 | 74 | ● | Before 71 | Longer than 12 |
| 5 | M | 77 | 68 | ● | 53 | 22 |
| 6 | F | 73 | 70 | × | － | － |
| 7 | M | 72 | 65 | × | － | － |
| 8 | M | 71 | 69 | ● | 69 | 2.5 |
| 9 | F | 74 | 72 | × | 74 (at the same time with pancreatic cancer) | 0 |
| 10 | F | 81 | 80 | ● | 72 | 8 |
| 11 | F | 84 | 79 | ● | 74 | 5 |
| 12 | F | 76 | 75 | × | － | － |
| 13 | M | 62 | 60 | × | － | － |
| 14 | M | 78 | 77 | ● | 59 | 18 |

M=Male, F=Female, ●＝yes, ×＝none
